# Case Report: Post-traumatic splenosis and potential pitfall for PSMA-PET

**DOI:** 10.3389/fnume.2023.1319952

**Published:** 2023-11-27

**Authors:** Marina Nearchou, Elizabeth Georgiou, Alexis Vrachimis, Konstantinos Ferentinos, Iosif Strouthos

**Affiliations:** ^1^ Department of Radiation Oncology, German Oncology Centre, University Hospital of the European University, Limassol, Cyprus; ^2^ Department of Nuclear Medicine, German Oncology Centre, University Hospital of the European University, Limassol, Cyprus

**Keywords:** ectopic splenic tissue, splenosis, false positive, 18F-PSMA PET/CT, 99mTc-sulphur colloid SPECT, prostate cancer

## Abstract

**Background:**

18F-prostate specific membrane antigen (PSMA) PET is fast becoming the gold-standard in prostate cancer, both in staging of intermediate-/high-risk patients and in re-staging patients with biochemical failure. Several pitfalls of 18F-PSMA PET have been reported, and we report, to our best of knowledge, for the first time, a case which could have been falsely diagnosed as peritoneal spread.

**Case presentation:**

A 67-year-old patient with high-risk prostate cancer underwent staging with 18F-PSMA-1007 PET/CT (PSMA-PET/CT). PSMA-PET/CT revealed a histologically confirmed prostatic malignancy in the peripheral left zone. Unexpectedly, additional multiple highly PSMA-expressing intraabdominal formations were discovered. Based on apparent anatomic asplenia and a history of traumatic splenic rapture during childhood, a suspicion of post-traumatic splenosis was raised. For further non-invasive evaluation, a C-99 sulphur colloid scintigraphy with SPECT was conducted, confirming the presence of multiple functional ectopic splenic tissues. This is, to our best of knowledge, the first case utilising 18F-PSMA-1007-PET/CT and 99mTc-sulphur colloid SPECT to detect intraabdominal splenosis, highlighting the high potential of nuclear medicine in such trivial cases.

## 1. Introduction

Prostate cancer (PCa) is the second most frequently diagnosed malignancy in men and the fifth leading cause of cancer death worldwide. Positron emission tomography (PET) imaging with 18F-prostate-specific membrane antigen (PSMA) ligands has been recently introduced into routine imaging practice of patients with PCa, assisting both in primary diagnosis and recurrencies (1). The spleen is acknowledged as a PSMA-expressing organ, presenting physiologic tracer uptake.

Ectopic spleen is considered a rare condition, which is widely believed to occur because of laxity or the absence of physiological ligaments (congenital or postnatal), consisting of the splenogastric, splenorenal, or splenocolic ligaments. Most cases are congenital, whereas acquired ectopic spleen may be a consequence of surgery or trauma. The clinical symptoms of splenosis are nonspecific and related to the ectopic site or the presence of complications (2, 3).

We report a case presenting with widespread PSMA-avid peritoneal deposits in a patient with a history of traumatic splenic rapture following remnant splenectomy in childhood and evident abdominal splenosis. We demonstrated this as a potential pitfall for 18F-PSMA-PET/CT, which cannot differentiate it from abdomino-peritoneal metastatic spread. A 99mTc-sulphur colloid SPECT study was used for non-invasive confirmation of the suspicion (4).

## 2. Case report

**Figure 1:**
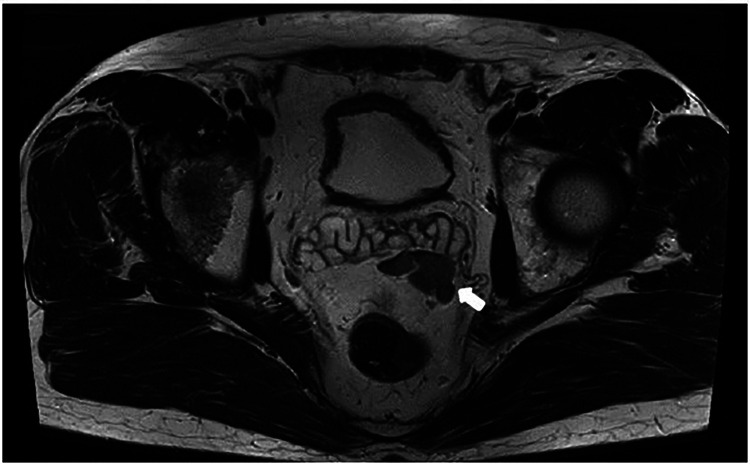
Axial mpMRI image showing suspicious mass in the anterior mid rectal fat.

A 67-year-old male patient with histologically proven PCa and a serum PSA value of 5.28 ng/ml was referred to our centre for further evaluation. A multiparametric prostate MR revealed a PI-RADS 5 lesion of the prostate gland and a highly suspicious mass of unknown origin in the mid anterior rectal fat, at the level of the left seminal vesicle (35 × 21 mm, Figure 1). A stereotactic MR-fusion biopsy of the prostatic gland and of the suspicious anterior mid rectal mass confirmed Gleason score 8 (4 + 4), Grade group 4, adenocarcinoma of the prostate and findings compatible with non-specific lymphoproliferative tissue in the suspicious mass.

**Figure 2:**
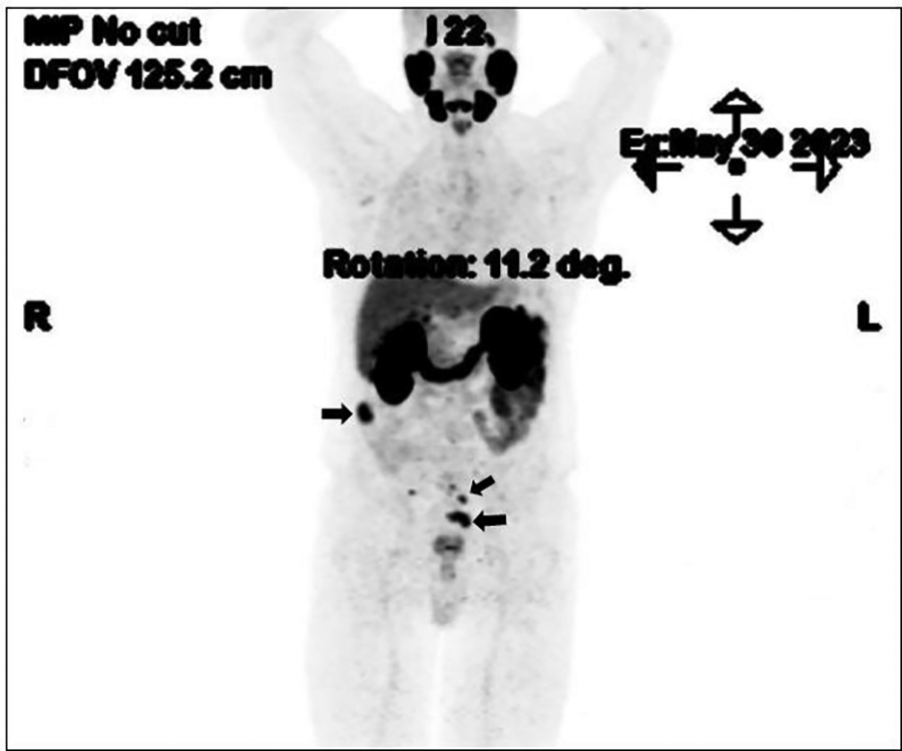
Maximum intensity projection view of PET/CT image.

Owing to the high-risk PCa profile and the suspicious mesorectal finding, we proceeded with 18F-PSMA-PET/CT for staging completion. In addition to uptake at the primary tumour site at the prostate gland, multiple foci of increased tracer up to an SUV peak of 19.2 (SUVmax 28.0) were detected, corresponding to multiple soft tissue intraabdominal masses (Figure 2). The absence of orthotopic spleen raised a suspicion of splenosis. The patient's history revealed a post-traumatic (partial) splenectomy 50 years ago and no significant or recurrent infections throughout his lifetime.

**Figure 3:**
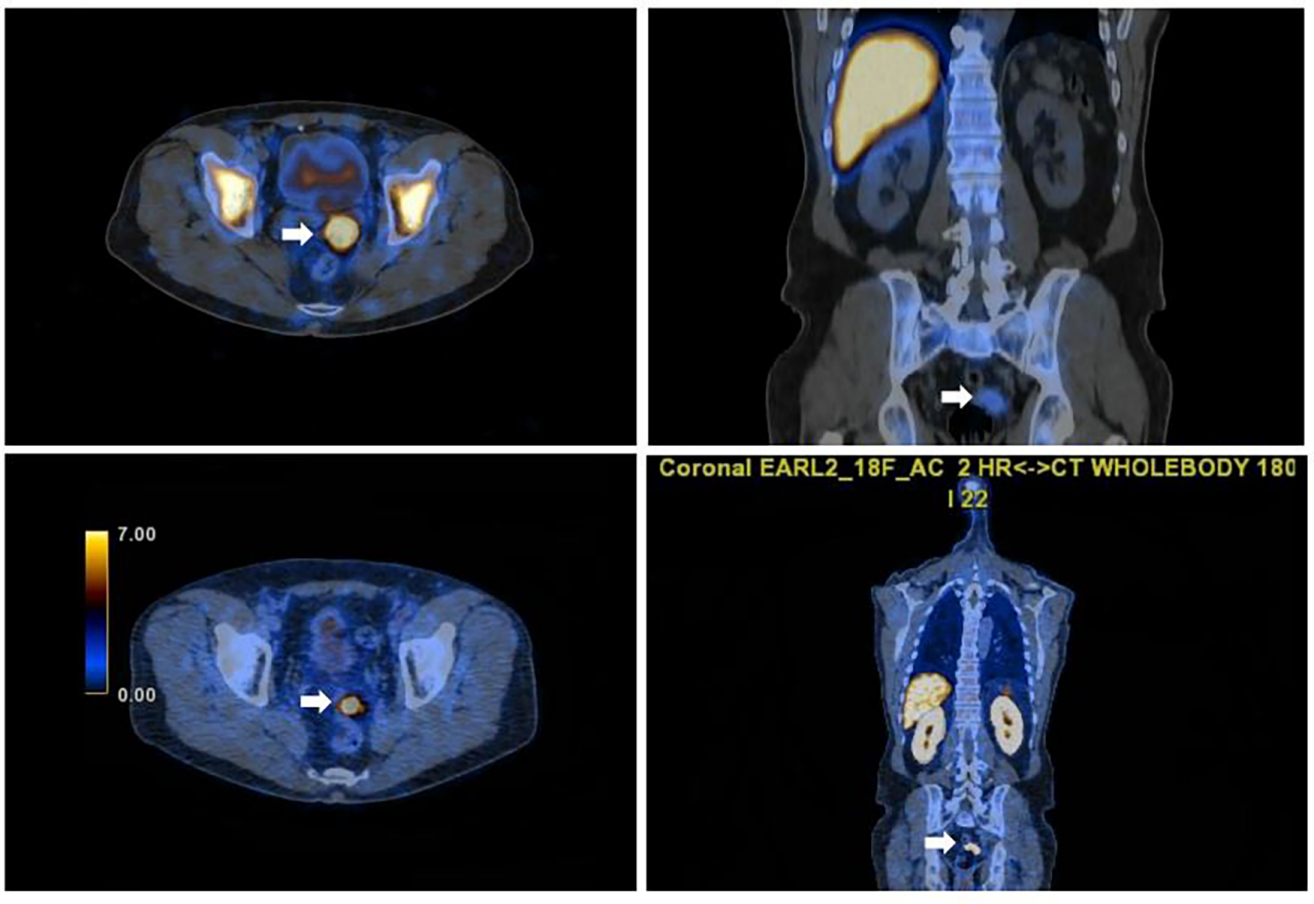
Axial and sagittal fused SPET/CT images of Tc-99m scan(left and right pictures) confirming functioning pelvic splenic tissue and concordant with the focal PSMA uptake seen on the PSMA PET/CT images (right and left lower images).

**Figure 4:**
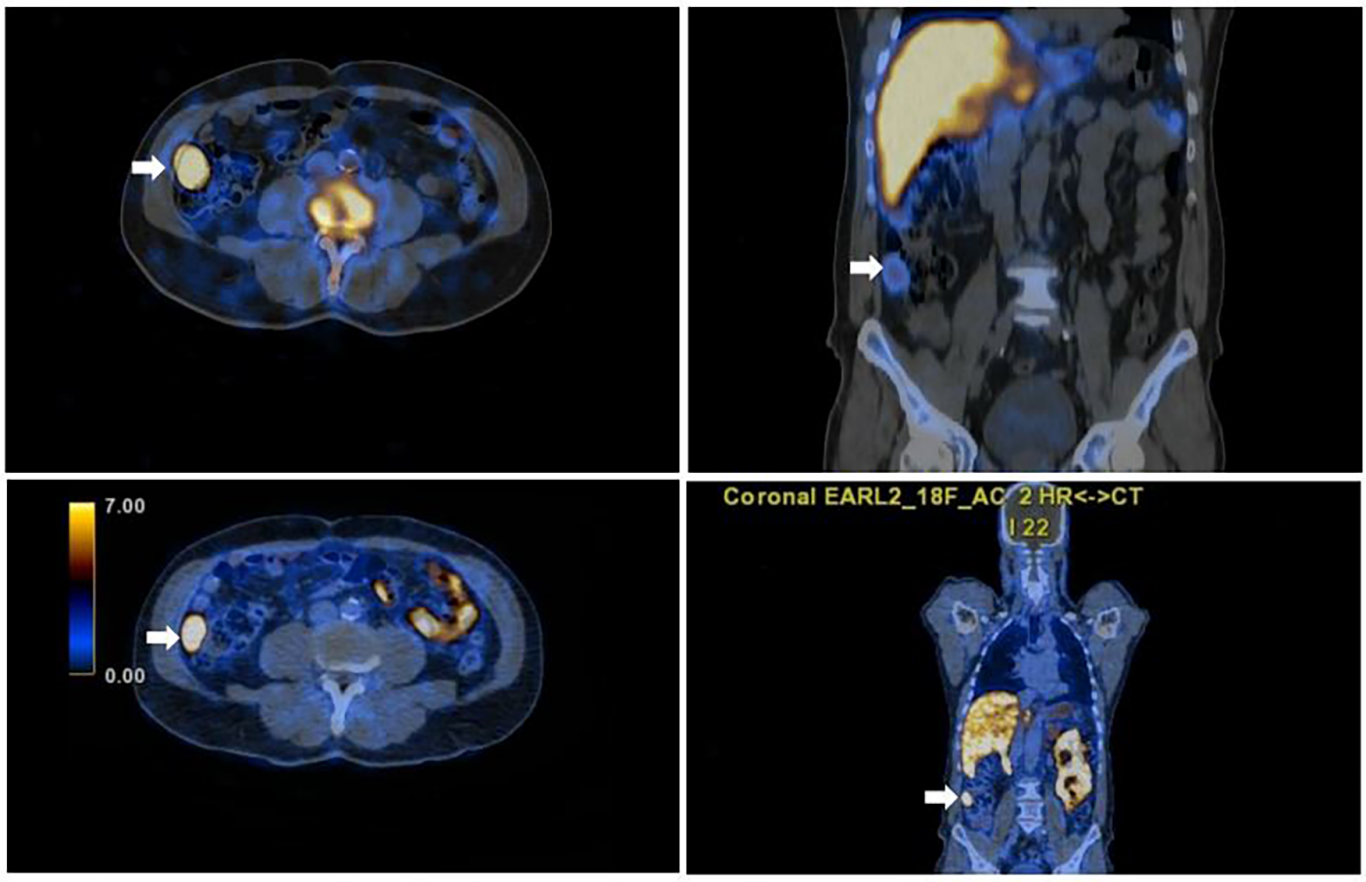
Axial and sagittal fused SPET/CT images of Tc-99m scan (left and right upper images) confirming functioning abdominal splenic tissue and concordant with the focal PSMA uptake seen on the axial and sagittal PSMA PET/CT images (right and left lower images).

A subsequent non-invasive clarification with Tc-99m sulphur using a hybrid SPECT/CT camera was performed. Full matching was observed between Tc-99m and 18F-PSMA uptake in all abdominal and pelvic lesions (Figures 3, 4), hence the diagnosis of splenosis was indirectly confirmed.

Regarding his oncological treatment, all the available treatment options were discussed, and the patient decided to proceed with prostatectomy followed by regularly checking of PSA values. Since the treatment, the patient's serum PSA value has remained stable at 0.01 ng/ml.

## 3. Discussion

Modern molecular imaging, such as PSMA-PET scanning, is advisable for staging patients with intermediate or high-risk PCa, and for restaging of all patients. In comparison with conventional imaging, such as CT and bone scintigraphy, the superiority of PSMA-PET is evident, both in terms of specificity and sensitivity, in detecting locoregional lymphatic and/or distant spread.

Correlation between imaging findings (usually CT and/or MRI), patient history, and the expected pattern of disease spread reinforces the distinction of lesions that are more likely related to PCa from those that could lead to misdiagnosis.

PSMA-expressing organs, such as the spleen, have physiologically elevated uptake on PET/CT. Therefore, in this case, the presence of ectopic splenic tissue could have been falsely diagnosed as metastatic disease/peritoneal carcinomatosis (3). Ectopic splenic tissue can be either an accessory congenital spleen or acquired splenosis. Splenosis can occur after splenic rupture via trauma or surgery and is usually found incidentally. Unless symptomatic, therapy is not indicated (5). Since histological confirmation of splenosis is limited, SPECT/CT is performed, which allows the diagnosis of ectopic splenic tissue as splenosis.

## 4. Conclusion

PSMA-positive abdomino-peritoneal lesions may be detected in the case of splenosis. This can lead to misinterpretation as metastatic spread. The patient's history, especially previous trauma or spleen related surgery and asplenia, should raise a suspicion of ectopic splenosis. 99m-Tc sulphur colloid or Tc-99m DRBC with SPECT/CT can offer further clarification, as biopsy is challenging for splenic tissue (4).

## Data Availability

The original contributions presented in the study are included in the article/Supplementary Material, further inquiries can be directed to the corresponding author.
